# Venous Thromboembolism in Cancer: Predictors of Recurrence and Bleeding in Patients on Direct Oral Anticoagulants

**DOI:** 10.3390/jcm14082752

**Published:** 2025-04-16

**Authors:** Alaa Shahbar, Afnan Noor, Alqassem Y. Hakami, Abdulfattah Y. Alhazmi, Bassim Albeirouti, Raghad Althubaiti, Khalid O. Alolasi, Mohammed Almazmumi, Faisal A. Alhamdan, Rawan A. Albariqi, Mohammed Alnuhait

**Affiliations:** 1 Pharmaceutical Practices Department, College of Pharmacy, Umm Al-Qura University, Makkah 24382, Saudi Arabia; 2 Pharmaceutical Care Department, King Faisal Specialist Hospital & Research Center, Jeddah 22234, Saudi Arabia; 3 King Abdullah International Medical Research Center, Jeddah 22384, Saudi Arabia; 4 College of Medicine, King Saud Bin Abdulaziz University for Health Sciences, Jeddah 21423, Saudi Arabia; 5 Section of Adult Hematology & TCT, Department of Oncology, King Faisal Specialist Hospital & Research Center, Jeddah 22234, Saudi Arabia; 6 Pharmaceutical Care Department, Ministry of the National Guard—Health Affairs, Jeddah 11426, Saudi Arabia

**Keywords:** VTE recurrence, cancer patients, DOACs, treatment, bleeding, Saudi Arabia

## Abstract

**Introduction:** Cancer patients are at a heightened risk of veous thromboembolism (VTE) and bleeding complications. Direct oral anticoagulants (DOACs) are increasingly used due to their oral administration and lack of routine monitoring. However, factors such as drug interactions, chemotherapy, tumor location, and renal function may influence their safety and efficacy. This study evaluates clinical predictors of VTE recurrence and bleeding outcomes in cancer patients on DOAC therapy. **Methods:** A multicenter retrospective cohort study included 160 adult cancer patients treated with DOACs for VTE. Data on demographics, clinical characteristics, and outcomes—including VTE recurrence, major bleeding, minor bleeding, and clinically relevant non-major bleeding (CRNMB)—were analyzed. Logistic regression identified predictors of outcomes, with significance set at *p* < 0.05. **Results:** At six months, VTE recurrence occurred in 7.5% of patients, while major bleeding and CRNMB were observed in 6.3% and minor bleeding in 8.8%. Decreased creatinine clearance (OR = 0.957, *p* = 0.024) and dexamethasone use (OR = 18.03, *p* = 0.031) were significant predictors of major bleeding. NSAID use (OR = 12.37, *p* = 0.009) increased CRNMB risk. Major bleeding at 12 months was significantly associated with recurrent VTE (χ^2^(1, N = 160) = 10.03, *p* = 0.002). **Conclusions:** DOACs are effective for VTE in cancer patients, but careful monitoring of renal function and dexamethasone use is essential due to increased bleeding risk. Caution is advised with NSAIDs in this population.

## 1. Introduction

Cancer patients are more likely to develop venous thromboembolism (VTE) due to hypercoagulopathy associated with patient, disease, and treatment-related factors. Factors such as age, tumor location, tumor stage, anti-angiogenic agents, chemotherapy, and intravascular devices are increasing the risk of VTE up to four to seven times in cancer patients in comparison with non-cancer patients. Also, cancer patients are at higher risk of bleeding than non-cancer patients. Many therapeutic agents, such as low molecular weight heparin (LMWH), unfractionated heparin (UFH), warfarin, fondaparinux, and direct oral anticoagulants (DOACs), are being used for the treatment of cancer-associated VTE [1,2]. DOACs are preferred over the other agents, as they do not need frequent monitoring and are administered orally, despite the bleeding risk and drug–drug interactions. Many randomized clinical trials and meta-analysis studies have shown a reduction in VTE recurrence with DOACs compared to LMWH and warfarin. However, the bleeding risk results of those studies were conflicting [1,2]. The first published DOAC studies included a low percentage of the cancer population with heterogeneous cancer types, locations, and stages [2]. The sub-analysis of cancer patients who received rivaroxaban in EINSTEIN-DVT and EINSTEIN-PE showed comparable safety and efficacy outcomes between rivaroxaban and warfarin with enoxaparin [3]. More studies came up later to study the safety and efficacy of different DOAC agents in the cancer population. For instance, edoxaban versus dalteparin was studied in the Hokusai-VTE cancer trial, showing the non-inferiority of edoxaban to dalteparin in a composite of recurrent VTE or major bleeding; however, the major bleeding outcome was higher in edoxaban, especially in patients with gastrointestinal malignancies [4].

On the other hand, apixaban versus dalteparin in cancer patients showed lower VTE recurrence (HR = 0.099, CI95%, 0.113–0.78), *p* = 0.028 [5]. The Caravaggio trial of apixaban versus dalteparin showed a similar result to the ADAM-VTE trial. Nine patients with gastrointestinal malignancies in each group of apixaban and dalteparin had a significant bleeding incidence [6]. Rivaroxaban is another DOAC that was evaluated in the SELECT-D trial, in which rivaroxaban significantly lowered VTE recurrence with no significant increase in major bleeding nor clinically relevant non-major bleeding (CRNMB) [7]. DOACs are the preferred agents for treating cancer-associated VTE in patients without gastrointestinal or gastroesophageal lesions, according to the National Comprehensive Cancer Network (NCCN), with category-1 for apixaban and edoxaban. Apixaban is assumed to be safer than edoxaban in patients with gastrointestinal or gastroesophageal lesions because of the CARAVAGGIO trial results, with a category-2B recommendation in the NCCN guideline [8]. The aim of our study is to evaluate the clinical factors associated with the efficacy and safety of DOACs in cancer patients with VTE.

## 2. Method

This multicenter retrospective cohort study aimed to determine risk factors associated with venous thromboembolism (VTE) recurrence, major bleeding, and clinically relevant non-major bleeding in cancer patients receiving direct oral anticoagulants (DOACs). The study population included adult cancer patients aged 18 years and older who were prescribed a DOAC for VTE treatment. Patients were excluded if they had a mechanical heart valve, bacterial endocarditis, or active bleeding conditions such as hemophilia or other disorders known to increase bleeding risk. Specific exclusion criteria also included a history of intraocular bleeding within six months prior to therapy, gastrointestinal bleeding or endoscopically confirmed ulcers within six months, recent head trauma or major trauma within one month, neurosurgery within two weeks, or major surgery within one week before initiating therapy. Additionally, patients receiving concomitant strong cytochrome P-450 3A4 inhibitors (e.g., HIV protease inhibitors or systemic ketoconazole) or inducers (e.g., rifampicin, carbamazepine, or phenytoin), as well as p-glycoprotein inhibitors or inducers, were excluded to prevent significant drug interactions that could impact outcomes.

### 2.1. Data Collection

Data for this study were obtained from two tertiary hospitals’ electronic health records spanning from 2016 to August 2023. The collected variables included demographic information such as age, sex, body mass index (BMI), presence of an intravascular device, cancer type, cancer stage, history of chemotherapy treatment, and prior surgeries. Clinical factors assessed comprised renal function parameters, including serum creatinine and creatinine clearance, as well as platelet count, international normalized ratio (INR), aspartate aminotransferase (AST), and alanine aminotransferase (ALT) levels. Renal function was assessed using creatinine clearance (CrCl), calculated using the Cockcroft–Gault equation as follows: CrCl (mL/min) = [(140 − age) × weight (kg)]/[72 × serum creatinine (mg/dL)], with a correction factor of 0.85 applied for female patients. Additionally, prior VTE history and concomitant medication use—including aspirin, clopidogrel, non-steroidal anti-inflammatory drugs (NSAIDs), dexamethasone, selective serotonin reuptake inhibitors (SSRIs), and selective norepinephrine reuptake inhibitors (SNRIs)—were documented. The type, dose, and duration of DOAC therapy were also recorded. Study outcomes were categorized into efficacy and safety measures. Efficacy was evaluated based on the occurrence of recurrent VTE at six and twelve months. Safety outcomes included major bleeding, clinically relevant non-major bleeding (CRNMB), and minor bleeding, all defined according to the International Society on Thrombosis and Haemostasis (ISTH) criteria. Major bleeding was defined according to established criteria as follows: (1) overt bleeding associated with either a ≥2 g/dL decrease in hemoglobin or transfusion of ≥2 units of packed red blood cells; life-threatening bleeding in critical anatomical sites including intracranial (encompassing intracerebral (hemorrhagic stroke), subarachnoid, or subdural hemorrhage), intraspinal, epidural, intraocular, retroperitoneal, or pericardial spaces; or fatal bleeding. Additionally, clinically significant musculoskeletal bleeding (intra-articular hemorrhage or intramuscular bleeding with compartment syndrome) qualified as a major bleeding event. Clinically relevant non-major bleeding is defined as overt bleeding not meeting the criteria for major bleeding but associated with medical intervention, unscheduled contact with a member of the healthcare team, or temporary cessation of study treatment. All other overt bleeding events that do not qualify as either major bleeding or CRNMB were considered minor bleeding. The follow-up period was extended to six and twelve months post-DOAC initiation, with continued monitoring for patients using DOACs beyond twelve months until cessation of therapy.

### 2.2. Statistical Analysis

All statistical analyses were conducted using IBM SPSS Statistics for Windows, version 26. Descriptive statistics were used to summarize the data. Continuous variables were presented as mean ± standard deviation (SD) or median with interquartile range (IQR), depending on the normality of distribution. Categorical variables were summarized as frequencies and percentages. Inferential statistics included chi-square tests of independence to examine associations between categorical variables, such as bleeding complications and VTE recurrence. Logistic regression analysis was performed to identify predictors of VTE recurrence and bleeding outcomes. A *p*-value of <0.05 was considered statistically significant.

### 2.3. Clinical Factors Influencing Study Outcomes

Multivariate logistic regression analyses assessed potential predictors of venous thromboembolism (VTE) recurrence, major bleeding, clinically relevant non-major bleeding (CRNMB), and minor bleeding. The models incorporated demographic characteristics (sex), clinical parameters (creatinine clearance), procedural factors (intravenous device use), medical history (prior VTE, gastrointestinal cancer), treatment-related variables (chemotherapy, recent surgery), and concomitant medications (aspirin, clopidogrel, non-steroidal anti-inflammatory drugs (NSAIDs), and dexamethasone). Additionally, bleeding outcomes were evaluated as time-dependent covariates in the VTE recurrence model to examine their potential role in predicting subsequent thrombotic events.

## 3. Results

### 3.1. Baseline Characteristics

This study evaluated 160 cancer patients receiving DOACs for VTE. The mean age of participants was 61.5 years, with an average body mass index (BMI) of 28.6 kg/m^2^. Females comprised 59.4% of the cohort, while males accounted for 40.6%. Apixaban (95%) was the most commonly prescribed anticoagulant. The most prevalent cancer types were breast cancer (21.9%), colon cancer (11.3%), and lymphoma (8.8%), with 40% of patients diagnosed with stage IV metastatic cancer.

Several risk factors affecting the efficacy and safety of DOAC therapy were identified. Prior VTE was reported in 38.8% of patients, and 27.5% had gastrointestinal cancers. Additionally, 47.5% were undergoing active chemotherapy, and 23.8% had undergone surgery within the past three months. Among concomitant medications that may interact with anticoagulant therapy and influence bleeding risk, dexamethasone (50%) was the most frequently used, followed by NSAIDs (16.9%), aspirin (13.8%), and clopidogrel (8.1%). Detailed baseline characteristics are presented in Table 1.

### 3.2. Recurrent VTE and Its Association with Bleeding Events

At six months of therapy, 12 patients (7.5%) experienced recurrent VTE. To assess the potential relationship between different types of bleeding and VTE recurrence, chi-square tests were conducted for major bleeding, minor bleeding, and CRNMB. The analysis revealed no statistically significant association between any bleeding type and VTE recurrence. Specifically, Pearson chi-square results showed *p*-values above the significance threshold, with major bleeding (χ^2^(1, N = 160) = 2.40, *p* = 0.121), minor bleeding (χ^2^(1, N = 160) = 0.003, *p* = 0.958), and CRNMB (χ^2^(10, N = 159) = 0.092, *p* = 0.762). These findings indicate that the presence of bleeding events within the first six months of DOAC therapy does not significantly correlate with an increased risk of VTE recurrence.

The logistic regression model assessing the other clinical and treatment-related factors associated with venous thromboembolism (VTE) recurrence at six months included 141 patients. Although the model demonstrated a stable fit, it lacked overall statistical significance (χ^2^ = 7.86, *p* = 0.796) and exhibited weak predictive power (Nagelkerke R^2^ = 0.129). The model correctly classified all non-recurrent cases but failed to identify any recurrent events, reflecting both the limited number of positive cases (*n* = 11) and the substantial class imbalance. No examined predictors—including sex, creatinine clearance, intravenous device use, chemotherapy exposure, recent surgery, or medication use—demonstrated statistically significant associations with recurrence. Notably, intravenous device use and recent surgical history showed elevated point estimates, though these associations did not reach statistical significance. These findings suggest the limited utility of the current model for predicting six-month VTE recurrence in this cohort.

By twelve months, five patients (3.12%) had recurrent VTE. Unlike the six-month analysis, a statistically significant association was identified between major bleeding and VTE recurrence (Fisher’s exact test, *p* = 0.032), with a moderate effect size (Phi = 0.25, *p* = 0.002). Additionally, chi-square results reinforced this finding (χ^2^(1, N = 160) = 10.03, *p* = 0.002). However, no significant associations were observed between VTE recurrence and minor bleeding (χ^2^(1, N = 160) = 0.81, *p* = 0.366) or CRNMB (χ^2^(1, N = 159) = 0.346, *p* = 0.556). These results suggest that major bleeding significantly predicts VTE recurrence at 12 months, while minor bleeding and CRNMB do not appear to impact recurrence rates. The findings underscore the importance of closely monitoring major bleeding events in patients on DOAC therapy, especially during prolonged treatment periods, to enhance patient outcomes.

The expanded analysis of twelve-month VTE recurrence for the other predictive clinical factors (*n* = 141) yielded a statistically significant overall model (χ^2^ = 22.95, *p* = 0.028) with moderate explanatory power (Nagelkerke R^2^ = 0.660). However, several methodological challenges emerged: failure to achieve convergence within 20 iterations, extreme coefficient estimates with inflated standard errors suggesting quasi-complete separation, and profound class imbalance (only four recurrence events). Despite high classification accuracy (98.6%), these issues raise concerns about model overfitting and interpretability. No individual predictors attained statistical significance, though creatinine clearance approached significance as a potential protective factor (*p* = 0.075). Several covariates—including recent surgery, chemotherapy exposure, and NSAID use—demonstrated clinically notable effect sizes without statistical significance.

### 3.3. Major Bleeding

Among the 160 patients included in this study, 10 patients (6.3%) experienced major bleeding, as defined as ISTH. To assess potential predictors of bleeding risk, various clinical factors, including cancer type, gender, anticoagulant use, history of VTE, chemotherapy, and concomitant medications, were analyzed. Among the predictors examined, creatinine clearance (CrCl) and dexamethasone use were found to be significantly associated with bleeding risk. CrCl exhibited an inverse relationship with bleeding risk (B = −0.044, *p* = 0.024), with an odds ratio (Exp(B)) of 0.957. This finding suggests that for every unit increase in CrCl, the odds of major bleeding decrease by approximately 4.4%, highlighting the crucial role of renal function in managing bleeding risk in patients receiving DOACs.

Conversely, dexamethasone use was associated with a significantly higher bleeding risk (B = 2.892, *p* = 0.031), with an odds ratio of 18.03. This indicates that patients receiving dexamethasone had substantially increased odds of experiencing major bleeding (Table 2).

Additionally, a comparison between gastrointestinal (GI) cancer and non-GI cancer groups was performed to determine any potential association with major bleeding. The results showed no statistically significant difference between the two groups (χ^2^(1, N = 160) = 0.84, *p* = 0.361), with an effect size (Phi) of −0.072 (*p* = 0.361). This indicates that GI cancer status by itself may not be a reliable independent predictor of major bleeding in patients undergoing DOAC therapy.

### 3.4. Minor Bleeding

Of 160 patients, 14 (8.8%) patients experienced minor bleeding based on the definition of ISTH. However, no variables were identified as statistically significant predictors of minor bleeding, as shown in Table 3. The omnibus test indicated that the overall model did not significantly predict the occurrence of CRNMB (chi-square = 10.907, df = 11, *p* = 0.451). Additionally, the model’s explanatory power was low, as demonstrated by Cox and Snell R^2^ (0.068) and Nagelkerke R^2^ (0.149), suggesting that the model accounted for only a small portion of the variance in minor bleeding.

When examining individual variables, none reached statistical significance. Although dexamethasone use was positively associated with minor bleeding (B = 0.573), this association did not reach statistical significance (*p* = 0.398). These findings suggest that no specific clinical or demographic factors were strong independent predictors of minor bleeding in this patient cohort.

### 3.5. CRNMB

Of the 160 patients included in the study, 10 (6.3%) experienced clinically relevant non-major bleeding (CRNMB). NSAID use emerged as a significant predictor of CRNMB (B = 2.515, *p* = 0.009), with an odds ratio of 12.37, indicating a substantially increased likelihood of bleeding in patients using NSAIDs.

However, other variables, including gender, prior VTE, intravenous (IV) devices, gastrointestinal cancer, chemotherapy status, recent surgeries, aspirin (ASA) use, clopidogrel, and dexamethasone, did not reach statistical significance (Table 4). These findings underscore the need for careful monitoring of NSAID use in patients receiving DOAC therapy, given its strong association with increased CRNMB risk.

## 4. Discussion

This study highlights key clinical factors influencing the efficacy and safety of DOACs in cancer patients with VTE. The recurrence rate of VTE at six months was 7.5%, aligning with retrospective real-world data reporting a similar recurrence rate.^9^ However, our cohort exhibited higher rates of major bleeding and clinically relevant non-major bleeding (CRNMB), both at 6.3%, compared to the retrospective study that reported lower rates of major bleeding (3.7%) and CRNMB (5.3%) [9].

In our study, the majority of patients received apixaban for VTE treatment. Notably, gastrointestinal (GI) cancer did not increase the risk of major, minor, or clinically relevant non-major bleeding. This finding aligns with the Caravaggio trial, which demonstrated that apixaban was not associated with a significant increase in bleeding compared to dalteparin.^6^ However, a prospective cohort study reported a higher major bleeding rate in patients with GI cancer compared to those without (15.59 vs. 3.26 per 100 person-years; *p* = 0.004) [10]. Notably, most GI cancers in our study were located in the lower gastrointestinal tract, which may explain the absence of an increased bleeding risk. Previous studies have shown that upper GI cancers pose a greater risk of DOAC-associated bleeding (22.4%) compared to lower GI cancers (15.4%) and non-GI cancers (11.6%) [11]. Additionally, after adjusting for confounding variables, the increased bleeding risk remained significant only for upper GI cancers (adjusted hazard ratio: 2.25; 95% CI, 1.31–3.87; *p* = 0.003) [11]. These results indicate that while apixaban continues to be a safe and effective choice for managing venous thromboembolism (VTE) in cancer patients, extra caution should be exercised when treating individuals with upper gastrointestinal (GI) malignancies.

Creatinine clearance (CrCl) demonstrated an inverse relationship with bleeding risk, a finding that contradicts prior studies such as the Caravaggio trial, which reported no increased risk of major bleeding in patients with mild to moderate renal impairment [12]. Similarly, a subgroup analysis of the ONC-DVT trial found no significant rise in bleeding risk among cancer patients with renal impairment (CrCl < 50 mL/min) treated with edoxaban compared to dalteparin [13]. This inverse relationship between CrCl and the risk of major bleeding has been shown in our population despite approximately 98% of our patients having CrCl ≥ 30 mL/min. While CrCl was a statistically significant predictor of bleeding risk (*p* = 0.024), the model’s explanatory power was limited, as reflected by Cox and Snell R^2^ = 0.131 and Nagelkerke R^2^ = 0.368, indicating that only 13.1% to 36.8% of the variance in bleeding outcomes was explained. Furthermore, the model exhibited high specificity (99.3%) but critically low sensitivity (11.1%), failing to identify most major bleeding cases. A sensitivity of 11.1% means that the model fails to identify nearly 90% of major bleeding cases, which raises concerns about its practical utility in clinical decision-making. Future research should investigate additional potential confounders to enhance the accuracy of bleeding risk models in cancer patients receiving DOAC therapy.

Dexamethasone is theoretically classified as a weak inducer of cytochrome P450 (CYP) 3A4 and P-glycoprotein (P-gp), which may enhance the metabolism of drugs metabolized through these pathways. Both apixaban and rivaroxaban are substrates of CYP3A4 and P-gp, raising the possibility that concomitant administration of dexamethasone could reduce their plasma concentrations and potentially compromise their efficacy [14]. However, in our study population, dexamethasone use was not associated with an increased risk of recurrent thromboembolic events, suggesting that this interaction may not be clinically significant in this context. To date, no randomized controlled studies have specifically investigated the interaction between dexamethasone and apixaban or rivaroxaban in cancer patients receiving these DOACs for the treatment of venous thromboembolism (VTE). Existing evidence primarily focuses on using these agents for VTE prophylaxis in high-risk populations, such as multiple myeloma patients receiving immunomodulators. For instance, combining immunomodulators and dexamethasone increases the risk of VTE by 5–8% [15]. In a single-arm pilot study involving 50 patients receiving this combination, prophylactic use of apixaban 2.5 mg twice daily was associated with no incidence of thromboembolism or major bleeding, suggesting a potential protective effect [15].

The increased risk of major bleeding associated with dexamethasone cannot be attributed to CYP3A4 and P-glycoprotein (P-gp) induction, as these mechanisms would typically reduce DOAC concentrations. Instead, the heightened bleeding risk may be explained by other factors, including impaired gastrointestinal mucosal repair and increased vascular fragility induced by dexamethasone [16,17].

Given that 95% of patients in our cohort received apixaban, the generalizability of findings to other DOACs is limited and should be interpreted with caution. Our study is the first to evaluate DOAC-associated risk factors for VTE recurrence, major bleeding, minor bleeding, and clinically relevant non-major bleeding (CRNMB) in Saudi cancer patients receiving VTE treatment. Although the sample size was relatively small, further randomized controlled trials are needed to identify additional predictive factors and potential confounders that may impact the safety and efficacy of DOAC therapy in this population.

## 5. Conclusions

This study assessed the efficacy and safety of DOACs in cancer patients with VTE, finding a 7.5% recurrence rate at six months and notable bleeding risks (major bleeding 6.3%, CRNMB 6.3%). Decreased creatinine clearance and dexamethasone use were significant predictors of major bleeding, while NSAIDs increased CRNMB risk. Other factors, including gastrointestinal cancer, aspirin, clopidogrel, IV device use, prior VTE, recent surgery, chemotherapy, and gender, had no significant impact. These findings highlight the need to monitor renal function and dexamethasone use carefully. Further studies are needed to refine bleeding risk models and optimize DOAC therapy in this high-risk group.

## Figures and Tables

**Table 1:** Baseline characteristics of study population.

Characteristics	Value
Age (years), mean ± SD	61.49 ± 13.26
BMI (kg/m^2^), mean ± SD	28.60 ± 7.16
Gender	
Male	65 (40.6%)
Female	95 (59.4%)
DOAC type	
Apixaban	152 (95.0%)
Rivaroxaban	8 (5.0%)
Cancer type	
Breast cancer	35 (21.9%)
Colon cancer	18 (11.3%)
Lymphoma	14 (8.8%)
Lung cancer	13 (8.1%)
Rectal cancer	10 (6.3%)
Ovarian cancer	10 (6.3%)
Endometrial cancer	10 (6.3%)
Pancreatic cancer	9 (5.6%)
Sarcoma	7 (4.4%)
Bladder cancer	6 (3.8%)
Renal cell carcinoma	4 (2.5%)
Multiple myeloma	4 (2.5%)
Hepatocellular carcinoma	3 (1.9%)
Thyroid cancer	2 (1.3%)
Prostate cancer	2 (1.3%)
CNS cancer	2 (1.3%)
Urothelial carcinoma	1 (0.6%)
others	10 (6.3%)
Cancer stage	
I	10 (6.25%)
II	12 (7.50%)
III	11 (6.87%)
IV	64 (40.0%)
Missing	18 (11.25%)
Prior VTE	62 (38.8%)
IV Device (2 weeks prior, during, after DOAC)	17 (10.6%)
Gastrointestinal cancer	44 (27.5%)
Metastasis to brain	9 (5.6%)
Active chemotherapy	76 (47.5%)
Any surgeries within 3 months	38 (23.8%)
Concomitant medications:	
ASA	22 (13.8%)
Clopidogrel	13 (8.1%)
NSAIDS	27 (16.9%)
SNRI/SSRI	12 (7.5%)
Dexamethasone	80 (50%)
Laboratory results:	
PLT (platelet/109/L) mean ± std	279.93 ± 142.242
Hgb (g/dL) mean ± std	10.99 ± 2.022
INR	1.153 ± 0.23
AST (unites/L) mean ± std	27.25 ± 26.72
ALT (unites/L) mean ± std	20.57 ± 17.7451
Crcl (mL/min) mean ± std	92.65 ± 45.09
CKD stage	
Stage 1	71(44.4%)
Stage 2	49 (30.6%)
Stage 3	37 (23.1%)
Stage 4	2 (1.3%)
Stage 5	1 (0.6%)

ALT: alanine aminotransferase; AST: aspartate aminotransferase; BMI: body mass index; chronic kidney disease; CrCl: creatinine clearance; Hgb: hemoglobin; INR: international normalized ratio; NSAIDs: non-steroidal anti-inflammatory drugs; PLT: platelets; SNRI/SSRI: serotonin–norepinephrine reuptake inhibitor/selective serotonin reuptake inhibitor.

**Table 2:** Logistic regression analysis of major bleeding risk in DOAC patients.

Risk Factor	B	SE	Wald	*p*-Value	Exp (B)
Gender (F ref)	−0.394	0.850	0.215	0.643	0.674
CrCl	−0.044	0.019	5.078	0.024	0.957
IV Device	1.080	1.042	1.075	0.300	2.946
Prior VTE	1.271	0.949	1.794	0.180	3.564
GI Cancer	−0.338	0.901	0.140	0.708	0.713
On chemotherapy	0.795	0.988	0.648	0.421	2.215
Surgery	1.058	1.015	1.085	0.297	2.880
ASA	1.156	1.237	0.873	0.350	3.177
Clopidogrel	−0.114	1.299	0.008	0.930	0.892
NSAIDS	0.089	1.023	0.008	0.931	1.093
Dexamethasone	2.892	1.343	4.637	0.031	18.031

CrCl, creatinine clearance; IV device, 2 weeks prior, during, after DOAC; surgery, any surgery within 3 months; ASA, acetylsalicylic acid (aspirin 100 mg or less).

**Table 3:** Logistic regression analysis of minor bleeding risk in DOAC patients.

Variable	B	S.E.	Wald	df	Sig.	Exp (B)
Gender	0.228	0.657	0.121	1	0.728	1.256
CrCl (mL/min)	−0.004	0.008	0.246	1	0.620	0.996
IV Device	1.436	0.832	2.978	1	0.084	4.204
Prior VTE	0.265	0.650	0.166	1	0.684	1.303
GI Cancer	−0.218	0.707	0.095	1	0.758	0.804
On Chemo	−1.410	0.761	3.434	1	0.064	0.244
Surgery	0.712	0.645	1.220	1	0.269	2.039
ASA	0.928	0.891	1.087	1	0.297	2.530
Clopidogrel	−19.847	10,472.74	0.000	1	0.998	0.000
NSAIDS	0.143	0.902	0.025	1	0.874	1.154
Dexamethasone	0.573	0.678	0.713	1	0.398	1.773

CrCl, creatinine clearance; IV device, 2 weeks prior, during, after DOAC; surgery, any surgery within 3 months; ASA, acetylsalicylic acid (aspirin 100 mg or less).

**Table 4:** Logistic regression analysis of CRNMB risk in DOAC patients.

Variable	B	S.E.	Wald	df	Sig.	Exp (B)
Gender	0.73	0.991	0.542	1	0.462	2.075
CrCl (mL/min)	−0.014	0.014	0.987	1	0.32	0.986
IV Device	1.734	0.968	3.21	1	0.073	5.663
Prior VTE	0.358	0.868	0.17	1	0.68	1.43
GI Cancer	−1.392	0.878	2.511	1	0.113	0.249
On Chemo	−0.278	1.032	0.073	1	0.787	0.757
Surgery	0.968	0.848	1.303	1	0.254	2.633
ASA	1.283	1.165	1.213	1	0.271	3.607
Clopidogrel	0.473	1.22	0.151	1	0.698	1.606
NSAIDS	2.515	0.956	6.925	1	0.009	12.37
Dexamethasone	−1.604	1.154	1.932	1	0.165	0.201

CrCl, creatinine clearance; IV device, 2 weeks prior, during, after DOAC; surgery, any surgery within 3 months; ASA, acetylsalicylic acid (aspirin 100 mg or less).

## Data Availability

All data supporting the findings of this study are available within the article.
